# An Antidote to Snake Poison

**Published:** 1881-12

**Authors:** 


### AN ANTIDOTE TO SNAKE POISON.

OUR doctors and men of science will be sadly remiss if they do not follow up the very promising experiments recently made by M. de Lacerda in the direction of a cure for snake-bite. He has found that permanganate of potash positively counteracts the poison of serpents. In a first series of experiments a solution of the poison was injected into the tissues of dogs, and its effects were, as usual, swellings, abscesses, loss of substance and destruction of tissue. When, however, one per cent. solution of permanganate of potash had been injected two minutes after the poison, these local injuries were quite obviated; there was merely a slight swelling where the syringe-point entered. Injection into the veins was then tried, and the permanganate agaia succeeded perfectly. In only two cases out of thirty did failure occur, the particular animals being young and badly fed; also the antidote was given at too long an interval after the poison, when the heart was already tending to stop. The permanganate, in one series of cases, was introduced half a minute after a solution of venom, and the dog showed no suffering at all beyond agitation and acceleration of the heart for a few minutes. In another series the characteristic mischief caused by the poison was allowed to manifest itself before the antidote was given. After two or three minutes, always in five, every trouble disappeared ; a slight general prostration followed for fifteen to twenty-five minutes, after which the animal would walk, and even run about, and resume its normal aspect. Other dogs poisoned similarly, but not receiving the antidote, died more or less quickly. Here is proof, well-nigh positive, that Condy's fluid, which contains the permanganate, might save countless lives in India and other snake-haunted lands. The reason seems suggested in M. Pasteur's late experiments—the poison-germs are oxygenated into harmlessness. Why, then, might not hydrophobia also yield up its terrible secret to this simple method? It ought to be tried ; but at all events no time should be lost by Indian doctors in repeating the process adopted by M. Lacerda with his dogs upon the victims of serpent-bite.—*London Daily Telegraph.*

---

IN the printed instructions for playing lawn tennis we observe the injunction, "First have a good racket." And this is the game countenanced by our best society.—*Lowell Citizen.*

